# Effect of azithromycin on incidence of acute respiratory exacerbations in children with HIV taking antiretroviral therapy and co-morbid chronic lung disease: a secondary analysis of the BREATHE trial

**DOI:** 10.1016/j.eclinm.2021.101195

**Published:** 2021-11-13

**Authors:** Amy Price, Grace McHugh, Victoria Simms, Robina Semphere, Lucky G Ngwira, Tsitsi Bandason, Hilda Mujuru, Jon O Odland, Rashida A Ferrand, Andrea M Rehman

**Affiliations:** 1 Department of Clinical Research, London School of Hygiene & Tropical Medicine, London, UK; 2 Biomedical Research and Training Institute, Harare, Zimbabwe; 3 MRC International Statistics and Epidemiology Group, Department of Infectious Disease Epidemiology, London School of Hygiene & Tropical Medicine, London, UK; 4 Department of Microbiology & HNTI, College of Medicine, Blantyre, Malawi; 5 Malawi-Liverpool-Wellcome Trust Clinical Research Programme, Blantyre, Malawi; 6 Department of Clinical Sciences, Liverpool School of Tropical Medicine, Liverpool, UK; 7 Faculty of Health Sciences, UiT, The Arctic University of Norway, Tromsø, Norway; 8 School of Health Systems and Public Health, Faculty of Health Sciences, University of Pretoria, Pretoria, South Africa

## Abstract

**Background:**

In the BREATHE trial weekly azithromycin decreased the rate of acute respiratory exacerbations (AREs) compared to placebo among children and adolescents with HIV-associated chronic lung disease (CLD) taking antiretroviral therapy (ART). The aim of this analysis was to identify risk factors associated with AREs and mediators of the effect of azithromycin on AREs.

**Methods:**

The primary outcome of this analysis was the rate of AREs by study arm up to 49 weeks. We analysed rates using Poisson regression with random intercepts. Interaction terms were fitted for potential effect modifiers. Participants were recruited from Zimbabwe and Malawi between15 June 2016 and 4 September 2018.

**Findings:**

We analysed data from 345 participants (171 allocated to azithromycin and 174 allocated to placebo). Rates of AREs were higher among those with an abnormally high respiratory rate at baseline (adjusted rate ratio (aRR) 2.08 95% CI 1.10-3.95 p-value 0.02) and among those with a CD4 cell count <200 cells/mm^3^ (aRR 2.71; 95% CI 1.27-5.76; p-value 0.008). We found some evidence for variation in the effect of azithromycin by sex (p-value for interaction=0.07); males had a greater reduction in the rate of ARE with azithromycin treatment than females. We found that azithromycin had a greater impact on reducing AREs in participants with chronic respiratory symptoms at baseline, those on 1st line ART, with a FEV_1_ score >-2 and participants without baseline resistance to azithromycin. However, there was no statistical evidence for interaction due to low statistical power.

**Interpretation:**

These may represent subgroups who may benefit the most from treatment with weekly azithromycin, which could help guide targeted treatment.

**Funding:**

There was no funding source for this post hoc analysis.

**Research in context**

**Evidence before this study**

At present little evidence exists on the management of chronic lung disease (CLD) in children and adolescents with HIV. We conducted a literature review of the clinical, lung function and radiological features of CLD in children and adolescents with Human immunodeficiency virus (HIV). We identified 17 studies, 16 observational and one randomised, placebo controlled trial which investigated the effect of prophylactic erythromycin on respiratory exacerbations in children with HIV-related bronchiectasis. This study found no difference in exacerbations between the treatment and placebo group, but only included 31 participants. Azithromycin has both anti-microbial and anti-inflammatory activity, which may help to suppress immune activation and provide prophylaxis against respiratory infections. In patients with cystic fibrosis, treatment with azithromycin resulted in improved lung function, reduced respiratory exacerbations and a reduced need for treatment with oral antibiotics. The BREATHE trial found that treatment with weekly azithromycin for 48 weeks did not result in improved lung function but did significantly lower the risk of acute respiratory exacerbations.

**Added value of this study**

In this *post hoc* analysis of the BREATHE trial we examined risk factors for acute respiratory exacerbations (AREs), and factors that modify the association between weekly azithromycin and risk of AREs. We found that a baseline abnormally high respiratory rate and CD4 count <200 cells/mm^3^ were associated with a higher risk of AREs. We found evidence that azithromycin was more effective at reducing AREs in male participants and in participants with chronic respiratory symptoms, an FEV_1_ Z-score ≥-2, those on 1^st^ line ART, and without resistance to azithromycin at baseline, although we were underpowered to detect statistical evidence for interaction in effects.

**Implications of all the available evidence**

These may represent subgroups who may benefit the most from treatment with weekly azithromycin. The use of targeted treatment may reduce concerns regarding antimicrobial resistance (AMR). Further studies to evaluate the sustainability of effect, the optimum dose and length of treatment are needed. These studies should also evaluate the risk of AMR and the cost-benefit of treatment.

Alt-text: Unlabelled box

## 1 Introduction

The scale-up of paediatric antiretroviral therapy (ART) has resulted in a dramatic increase in survival such that children who would have died in infancy or early childhood are now surviving to adolescence. However, children and adolescents growing up with HIV experience a range of multisystem comorbidities despite ART, which may be sequelae of infections that occur as a result of immunosuppression caused by HIV or a consequence of HIV infection itself or its treatment. Chronic lung disease (CLD) is one of the most common comorbidities among older children and adolescents with HIV [1]. In the pre-ART era, lymphoid interstitial pneumonitis (LIP) was the most common cause of CLD. LIP responds well to ART and is now a rare presentation to clinical practice [2]. Recent studies from sub-Saharan Africa (SSA) have shown that constrictive obliterative bronchiolitis is now the predominant underlying cause for CLD in children and adolescents with HIV, and is associated with morbidity including chronic cough, hypoxia, reduced exercise tolerance and recurrent respiratory tract infections [1,3].

The BREATHE (Bronchopulmonary function in response to azithromycin treatment for chronic lung disease in HIV-infected children) trial investigated whether adjuvant treatment with weekly azithromycin (AZM) for 48 weeks results in improved lung function in children and adolescents aged 6-19 years with HIV taking ART who had CLD [4]. Azithromycin did not lead to a significant improvement in forced expiratory volume in 1 second (FEV_1_) Z-score (primary outcome), but did lead to a significant reduction in the risk of acute respiratory exacerbations (AREs) (secondary outcome) [5]. Respiratory infections have been shown to be the most common cause of hospital admissions amongst adolescents with HIV established on ART, and AREs are likely to impact significantly on morbidity and quality of life [6].

In this *post hoc* analysis of the BREATHE trial, we investigated the effect of azithromycin on AREs to identify potential subgroups who would most benefit from azithromycin treatment. Specifically, we investigated the risk factors for AREs in both the placebo and azithromycin group; crude rates of AREs and factors that may modify the effect of azithromycin on risk of AREs.

## 2 Methods

### 2.1 Study Design

The BREATHE trial is a multicentre, individually randomised, placebo-controlled trial. A detailed study protocol has been published [4].

Participants were recruited from outpatient HIV clinics at the Harare Children´s Hospital (Zimbabwe) and Queen Elizabeth Central Hospital in Blantyre (Malawi). For those aged <18 years consent was sought from the guardian with age-appropriate assent from the participant, whilst those aged ≥18 years consented independently.

Ethical approval was provided by College of Medicine Research Ethics Committee (COMREC) (Malawi), the Medical Research Council of Zimbabwe and the Biomedical Research and Training Institute IRB (Zimbabwe), the London School of Hygiene and Tropical Medicine Ethics Committee (UK) and the University of Tromso Ethics Committee (Norway). The trial is registered with ClinicalTrials.gov, NCT02426112. This secondary analysis was conducted in adherence with the STROBE guidelines.

### 2.2 Participants

Participants were eligible if they were aged 6-19 years, had been on any combination of ART for at least six months and had CLD, defined as an FEV_1_ Z-score less than -1.0 with no reversibility (<12% improvement in FEV_1_ after 200mcg of salbutamol inhaled via a spacer), established by spirometry. Exclusion criteria included having a potentially fatal condition, tuberculosis (TB) or an acute respiratory infection (ARI) at the time of screening, pregnancy, breastfeeding, history of a cardiac arrythmia, a prolonged QTc interval (>440 milliseconds in males and >460 milliseconds females), creatinine clearance <30 mls/minute, elevated alanine aminotransferase (ALT) >2 times the upper limit of normal, known hypersensitivity to a macrolide and concomitant use of drugs known to cause QTc prolongation. TB screening was performed using the Xpert™ MTB/RIF (Cepheid, Sunnyvale, CA, USA) on one sputum sample obtained either spontaneously or through induction. Participants over 18 consented independently. For those aged <18 consent was sought from the guardian with age-appropriate assent from the participant.

### 2.3 Randomisation and Masking

Participants were randomised 1:1 by block randomisation to receive either an oral weekly weight-based dose of azithromycin or placebo. An independent statistician who had no involvement with the trial generated the randomisation schedule and allocation list using Stata version 14.0 (StatCorp, Texas, USA). Randomisation was performed with block sizes between two to six participants and was stratified by country. Participants and data collectors and outcome assessors were blinded to group allocation. The allocation list was sent directly to the study pharmacists, who prepared the study medication. The pharmacists were blind to treatment allocation but unblinded to group allocation as they were provided with allocations for study numbers into trial arm 1 or 2, and matched this to study medication labelled trial arm 1 or 2.

### 2.4 Procedures

Weight-based oral azithromycin tablets (10–19.9 kg, 250 mg; 20–29.9 kg, 500 mg; 30–39.9 kg, 750 mg; > 40 kg, 1250 mg) or identical placebo tablets were given weekly under direct observation by a treatment monitor identified within the family for a total of 48 weeks. Characteristics recorded at baseline include socio-demographic and clinical history, symptom history, drug history, spirometry, shuttle walk test, height, weight, electrocardiogram (ECG), serum creatinine and ALT, pregnancy test, sputum sample for TB screening, CD4 count, HIV viral load (VL). Participants were followed up at two weeks and at three monthly intervals thereafter for a total of 49 weeks. Participants were asked to attend for an unscheduled visit if they developed acute symptoms. AREs were defined as new or worsening respiratory symptoms (cough with or without sputum production, breathlessness, chest pain) with or without fever as assessed by a clinician. For participants attending with AREs, sputum and nasal swabs were taken and participants were treated with co-amoxiclav for 10 days. If this resulted in no improvement than a CXR and culture for *Mycobacterium tuberculosis* (M. tb) was performed. Participants were also asked to contact the study team if they were admitted to hospital, and were asked about hospitalisation at each study visit.

Detailed microbiological procedures and results have been reported previously [7,8]. Conventional culture was performed for common bacterial pathogens at baseline, 12 and 18 months on sputum and nasopharyngeal samples. Antibiotic susceptibility testing was performed on relevant cultured isolates using Vitek-2 (bioMerieux, France) or disk diffusion testing

### 2.5 Statistical Analysis

Statistical analysis was performed using STATA software version 16.1 (STATA Corp, College Station, Texas, USA). The pre-specified analysis of the BREATHE trial has been described elsewhere [4,5]. In this *post-hoc* analysis, incidence rate ratios (IRR) of AREs by arm with 95% confidence intervals (CIs) were calculated. Poisson regression was used to create a model of the effect of azithromycin on AREs, with random effects to account for multiple events within a participant. Lexis expansion was used to allow for joint adjustment of time variables (including season, calendar time and follow-up time), to look for variation of rates of AREs within these. Season was defined as rainy (November to April) and dry (May to October); calendar time was split into two groups, 2016-2017 versus 2018-2019; and follow-up time was split into four 12-week periods corresponding to the prescribing regimen. The relationship between ARE and time variables independent of other risk factors was explored. Collinearity among time variables was assessed by examining the change in standard errors between unadjusted and models adjusted for other time variables. All subsequent models were adjusted for those time variables which had Wald p-value <0.1 once adjusted for each other and where they did not exhibit co-linearity. Participants were censored at death, last study visit if lost to follow-up, date of withdrawal if withdrawn from the study or at 49 weeks after commencing study medication. Two participants missing HIV VL at baseline were excluded from all analyses.

Risk factors for AREs were examined among participants in both the azithromycin and placebo groups. Risk factors at enrolment included FEV_1_ Z-score, CD4 cell count, being on 2^nd^ line ART, weight-for- age Z-score, height-for-age Z-score [9], presence of current cough, tachypnoea (defined as a respiratory rate greater than the 99^th^ percentile of age-specific values) [10] and a history of treatment for TB. An adjusted model included trial arm, age group, sex, site and HIV VL at enrolment, in alignment with the primary analysis, as the latter characteristics were found to differ between trial arms by chance and were also associated with loss to follow-up [5]. The model estimates therefore accounted for missing data under a missing at random assumption. A second model was created which included the above mentioned characteristics as well as additional variables that were found to be associated with AREs in the initial model (p-value <0.1) (model 2).

Data from both trial arms was used to explore whether the reduced rate of AREs among those receiving azithromycin treatment, a pre-specified secondary outcome of the trial reported elsewhere [5], exhibited heterogeneity in effect by any of the risk factors assessed for their association with ARE. Adjusted random effects Poisson regression models were used to explore whether the effect of azithromycin on AREs was modified by baseline risk factors including age group, sex, FEV_1_ Z-score, CD4 cell count, HIV VL, history of treatment for TB, weight-for-age Z-score, height-for-age Z-score, presence of cough and tachypnoea, and additionally carriage of azithromycin-resistant bacteria (in sputum or from nasal swab), and time variables (season, calendar time and time in study). P-values for interaction were determined using a likelihood ratio test (LRT) and indicated the strength of evidence that the treatment effect of azithromycin versus placebo was heterogeneous between risk factor levels. Age was regrouped in the interaction term into two levels (6-15 and 16+) but adjusted as three levels in other models (6-10, 11-15 and ≥16). Viral load was adjusted for as a continuous variable but included in the interaction term as two levels, supressed (<1000 copies/ml) or unsuppressed (≥1000 copies/ml). Models were adjusted for *a priori* factors as per the primary analysis and time variables as previously stated. For risk factors where heterogeneity between the stratum specific estimates was observed unadjusted cumulative event curves, stratified by risk factor level and trial arm were generated. As AREs can occur multiple times in the same individual, so that individuals remain at risk of another ARE after they have recovered, participants who experienced an ARE were retained in the at-risk group and not censored unless they were lost to follow-up or died.

### 2.6 Role of the Funding Source

The BREATHE trial was funded by the GLOBVAC Programme of the Medical Research Council of Norway. The funder had no role in study design, data collection, data analysis, data interpretation or manuscript writing. There was no funding source for this post hoc analysis.

## 3 Results

Participants were recruited between 15 June 2016 and 4 September 2018 and follow up ended in August 2019. Of the 347 participants included in the BREATHE trial, 345 participants had no missing information for all variables of interest and were included in this analysis, with 171 participants randomised to the azithromycin and arm and 174 participants randomised to the placebo arm. Baseline characteristic of participants stratified by trial arm and outcome (at least one ARE episode) are summarised in Table 1. In both trial arms, those who experienced at least one ARE were older, had lower FEV_1_ Z-score at baseline, had a lower CD4 cell count, higher HIV VL, and commenced ART at older age.

**Table 1:** Baseline characteristics of participants by trial arm and whether they experienced at least one ARE.

	All participants	Azithromycin (AZM) arm	Placebo arm		
		No ARE	At least one ARE	No ARE	At least one ARE
Total, n (%)	345	155 (90.6)	16 (9.4)	144 (82.8)	30 (17.2)
Median (IQR) age in years	15.3 (12.7-17.7)	14.7 (12.2-16.9)	15.2 (14.2-17.0)	15.1 (13.0-18.10)	16.1 (12.7-18.6)
Female sex, n (%)	168 (48.7)	67 (85.9)	11 (14.1)	76 (84.4)	14 (15.6)
Site: Zimbabwe, n (%)	241 (69.9)	106 (88.3)	14 (11.7)	95 (78.5)	26 (21.5)
Mean FEV_1_ Z-score at enrolment (SD)	-2.0 (0.8)	-2.0 (0.7)	-2.4 (0.8)	-1.9 (0.7)	-2.3 (1.0)
Mean FEV_1_ in litres at enrolment (SD)	1.7 (0.5)	1.6 (0.5)	1.5 (0.3)	1.7 (0.5)	1.7 (0.6)
Mean FVC Z-score (SD)	-1.7(0.9)	-1.8 (1.0)	-2.0 (1.0)	-1.6 (0.78)	-2.1(1.2)
Mean FVC in litres (SD)	2.0(0.6)	2.0 (0.6)	1.8 (0.4)	2.0 (0.6)	2.1(0.7)
Mean FEV1/FVC ratio Z-score (SD)	-0.7 (1.1)	-0.7(1.13)	-1.1 (1.1)	-0.7 (1.1)	-0.8 (1.3)
Mean FEV1/FVC ratio (SD)	0.8 (0.8)	0.9 (0.8)	0.8 (0.8)	0.8 (0.1)	0.8 (0.1)
Median (IQR) CD4 count cells/mm3	572 (356-784)	611 (418-788)	566.5 (360.5-856)	561.5 (343-807.5)	456 (248-659)
Median (IQR) HIV VL copies/ml	400 (39-13200)	309 (39-17700)	398 (59.5-2200)	286.5 (42-11180)	1835 (400-52700)
No (%) with HIV VL <1000 copies/ml	194 (56.2)	91 (91.0)	9 (9.0)	81 (86.2)	13 (13.8)
Mean (SD) duration of ART use in years No (%) Missing	6.3 (3.2) 11 (3.2)	6.4 (3.3) 4 (2.5)	6.7 (2.8)	6.3 (3.2) 5 (3.5)	6.2 (3.1) 2 (6.7)
Mean (SD) age in years at ART initiation No (%) Missing	8.6 (4.0) 10 (2.9)	8.1 (4.2) 3 (1.9)	8.6 (3.2)	8.8 (3.7) 5 (3.5)	10.1 (3.4) 2 (6.7)
No (%) on second line ART regimen	87 (25.5)	38 (82.6)	8 (17.4)	31 (75.6)	10 (24.4)
Mean (SD) weight-for-age Z-score	-2.2 (1.5)	-2.2 (1.4)	-2.3 (1.5)	-2.1 (1.5)	-2.2 (1.3)
No (%) underweight	180 (52.2)	87 (89.7)	10 (10.3)	68 (81.9)	15 (18.1)
Mean (SD) height-for-age Z-score	-2.1 (1.2)	-2.1 (1.2)	-2.4 (1.1)	-2.1 (1.3)	-1.9 (1.1)
No (%) stunted	174 (50.4)	84 (89.4)	10 (10.6)	67 (83.8)	13 (16.3)
No (%) with history of treatment for TB	97 (28.2)	50 (86.2)	8 (13.8)	30 (76.9)	9 (23.1)
No (%) admitted for chest problems in the past 12 months	6 (1.73)	2 (100.0)	0 (0)	1 (33.3)	2 (66.7)
No (%) with cough	31 (9.0)	12 (92.3)	1 (7.7)	10 (55.6)	8 (44.4)
Mean (SD) respiratory rate	22.4 (3.1)	22.1 (3.0)	23.2 (3.4)	22.5 (3.3)	23 (3.0)
No (%) with abnormal RR	151 (43.8)	59 (89.4)	7 (10.6)	64 (75.3)	21 (24.7)
No (%) with resistance to AZM	34 (9.9)	12 (85.7)	2 (14.3)	18 (90.0)	2 (10.0)

In the azithromycin arm, more females developed at least one ARE compared to males (68.8% vs 31.2%), but this was not the case in the placebo arm where a similar proportion of females and males developed at least one ARE (46.7% vs 53.3% respectively). Participants who in the azithromycin arm experienced at least one ARE were more malnourished than those in the placebo arm who experienced at least one ARE. Overall, 9.0% of participants had a cough at baseline; however, 26.7% of the participants in the placebo group who developed an ARE had a cough at baseline. An abnormal respiratory rate at baseline was observed in almost half of participants (43.8%), and this subgroup was also overrepresented in those who developed one or more AREs in the placebo arm, with 70.0% of these participants having an abnormal respiratory rate at baseline (Table 1).

There were 38 episodes of AREs amongst placebo group participants and 19 total episodes of AREs in the azithromycin arm, over a total of 154 and 157 person years respectively. In the azithromycin arm 14 participants had 1 ARE, 1 had 2 AREs and 1 had 3. In the placebo arm 24 participants had 1 ARE, 4 had 2 AREs and 2 had 3. In total 37 out of 57 AREs (64.9%) were treated with antibiotics. There was evidence that the rate of AREs varied by season, calendar period and time in study in an unadjusted analysis (Tables S1 and S2). After adjusting for each other, season and calendar period remained associated with the rate of AREs both in the placebo arm (Wald p-value=0.04 and p=0.05 respectively) and in the azithromycin arm (Wald p-value= 0.04 and p=0.01). Rates of ARE were lower in the rainy season compared to the dry season and reduced over time (lower in 2018-19 compared to 2016-17). As a result, all subsequent analyses were adjusted for season and calendar time.

Our analysis found that site, FEV_1_ Z-score, baseline CD4 cell count, HIV VL, presence of a cough and abnormal respiratory rate were associated with the rate of AREs (Table 2, p-values <0.1) after adjusting for age, sex, site, and baseline VL. Effects were consistent after adjusting for the potential confounding effect of the identified risk factors, with the exception of baseline cough and HIV VL. There was evidence of a lower rate of AREs in participants in Malawi compared to those in Zimbabwe (fully adjusted RR 0.31; 95% CI 0.13-0.73; p-value 0.003). Having an FEV_1_ Z-score <-2 was found to be associated with a higher rate of AREs in the minimally adjusted model, although this result was attenuated in model 2 (fully adjusted RR 1.79 95% CI 0.98-3.27 p-value 0.06). Having a CD4 count <200 cells/mm^3^ was associated with increased risk of AREs (fully adjusted RR 2.71; 95% CI 1.27-5.76; p-value 0.008). Compared to those with a normal respiratory rate at baseline, those with an abnormal respiratory rate had a higher rate of AREs (fully adjusted RR 2.08 95% CI 1.10-3.95 p-value 0.02). Presence of a cough at baseline was associated with higher rates of ARE in minimally adjusted analysis (model 1), but this association was attenuated in the fully adjusted model indicating confounding (fully adjusted RR 1.26 95% CI 0.56- 2.84 p-value 0.58) (Table 2).

**Table 2:** Risk factors for AREs.

	Variable categories	Total episodes of AREs/100 person-years	Model 1 RR (95% CI) [1]	p-value[2]	Model 2 RR[3] (95% CI)	p-value[2]
Total		57/309				
Age (years)	6-15	30/182	1	0.90	1	0.25
	16+	27/127	0.96 (0.53-1.76)		0.69 (0.37-1.31)	
Sex	Male	25/161	1	0.25	1	0.47
	Female	32/148	1.41 (0.78-2.53)		1.29 (0.72-2.29)	
Site	Zimbabwe	50/216	1	0.005	1	0.003
	Malawi	7/93	0.33 (0.14-0.77)		0.31 (0.13-0.73)	
FEV_1_ Z-score	≥-2	21/168	1	0.03	1	0.06
	<-2	36/141	2.18 (1.16-4.10)		1.79 (0.98-3.27)	
CD4 count (cells/mm^3^)	≥ 200	43/279	1	0.006	1	0.008
	<200	14/30	3.00 (1.42-6.40)		2.71 (1.27-5.76)	
HIV VL (copies/ml)	<1000	25/176	1	0.09	1	0.50
	≥1000	32/133	1.67 (0.93-3.00)		1.30(0.70-2.47)	
ART line	1st	35/233	1	0.16	1	0.46
	2nd	22/76	1.59 (0.84-3.01)		0.90 (0.44-1.88)	
Weight for age Z-score	Not underweight	29/144	1	0.82	1	0.91
	Underweight	28/165	1.08 (0.57-2.04)		0.97 (0.52-1.79)	
Height for age Z-score	Not stunted	26/157	1	0.70	1	0.99
	Stunted	31/152	0.89 (0.48-1.64)		1.00 (0.55-1.82)	
Presence of a cough	No	45/282	1	0.04	1	0.58
	Yes	12/27	2.36 (1.07-5.19)		<	
Abnormal RR	No	21/174	1	0.02	1	0.02
	Yes	36/135	2.19 (1.14- 4.19)		2.08 (1.10- 3.95)	
History of TB	No	36/219	1	0.68	1	0.92
	Yes	21/89	1.15 (0.61-2.17)		0.97 (0.53-1.78)	
Season	Rainy	21/156	1	0.06	1	0.12
	Dry	36/153	1.67 (0.97-2.86)		1.67 (0.98-2.87)	
Calendar Period (years)	2016-2017	35/121	1	0.004	1	0.05
	2018-2019	22/188	0.44 (0.25-0.77)		0.49 (0.28-0.89)	

^1^adjusted for trial arm, age, sex, site, season, calendar time, and HIV VL (continuous)

^2^p-value from LRT

^3^adjusted for trial arm, age, sex, site and season, calendar time, HIV VL (continuous), abnormal RR, cough, CD4 count and FEV_1_ Z-score.

Stratum-specific ARE rates varied by trial arm and risk factor. However, statistical testing did not indicate heterogeneity in the effect of azithromycin versus placebo in the rate of ARE for risk factors measured at baseline (Table 3; Figure S1-S6). There was weak evidence that the effect of azithromycin versus placebo differed by sex (LRT test for interaction= 0.07), with a reduced rate of AREs with azithromycin treatment compared to placebo among males (RR 0.23 95% CI 0.08-0.66) but not among females (RR 0.77 95% CI 0.35-1.68). Lower rates of AREs with azithromycin treatment compared to placebo were found among those with a cough and an abnormal respiratory rate, compared to those without these symptoms, those on 1^st^ line versus 2^nd^ line ART, those without carriage of azithromycin resistant bacteria, and those with better baseline lung function (FEV_1_ Z-score ≥-2 vs <-2). However these findings did not reach statistical significance (Table 3).

**Table 3:** Rates of ARE stratified by arm and explanatory variables and stratum specific estimates of effect of azithromycin on total episodes of AREs.

Explanatory variable	Placebo arm		Azithromycin arm				
	Total episodes of AREs ^1^	Rate of total AREs[2] (95 % CI)	Total episodes of AREs [1]	Rate of total AREs[2] (95 % CI)	Adjusted RR for effect of azithromycin on total AREs [3] (95% CI)	p- value for interaction[4]	
Total	38/ 154	24.66 (17.29-36.44)	19/155	12.27 (7.42-21.83)	0.50 (0.29-0.87)		
Age (years)	6-15	17/82	20.79 (12.00-39.39)	13/100	12.93 (6.92-27.10)	0.60 (0.27-1.33)	0.4
	16+	21/72	29.0 (18.23-49.17)	6/54	11.04 (4.51-34.61)	0.35 (0.13-0.94)	
Sex	Male	20/76	26.44 (16.37-45.64)	5/85	5.88 (2.55-16.88)	0.23 (0.08-0.66)	0.07
	Female	18/78	22.95 (13.50-42.28)	14/70	20.04 (10.90-41.08)	0.77 (0.35-1.68)	
Site	Zimbabwe	33/107	30.78 (21.11-46.70)	17/108	15.68 (9.17-29.18)	0.49 (0.25-0.93)	0.85
	Malawi	5/47	10.67 (3.80-41.84)	2/47	4.30 (0.96-40.09)	0.41 (0.07-2.24)	
FEV_1_ Z-score	≥-2	17/84	20.21 (11.28-39.96)	4/84	4.79 (1.85-16.53)	0.25 (0.08-0.77)	0.17
	<-2	21/70	30.01 (19.41-49.03)	15/71	21.04 (11.82-41.29)	0.62 (0.30-1.30)	
CD4 count (cells/mm^3^)	<200	9/16	57.01 (25.75-151.80)	5/15	34.37 (7.68-321.78)	0.53 (0.14-1.98)	0.84
	≥200	29/138	20.97 (14.16-32.44)	14/140	9.98 (6.16-17.28)	0.46 (0.23-0.90)	
HIV VL (copies/ml)	<1000	16/83	19.22 (11.05-36.58)	9/92	9.73 (5.33-19.82)	0.49 (0.21-1.14)	0.98
	≥1000	22/71	31.06 (19.54-52.45)	10/62	16.02 (7.18-43.44)	0.50 (0.21-1.16)	
ART line	1st	26/119	21.78 (13.95-36.02)	9/113	7.95 (4.00-18.20)	0.35 (0.16-0.79)	0.32
	2nd	12/35	34.56 (19.42-67.44)	10/42	24.00 (11.49-58.94)	0.67 (0.25-1.77)	
Weight for age Z-score	Underweight	18/75	24.04 (14.53-42.80)	10/90	11.09 (6.29-21.51)	0.44 (0.19-1.03)	0.81
	Not underweight	20/79	25.25 (15.27-44.90)	9/65	13.90 (5.76-42.62)	0.51 (0.21-1.22)	
Height for age Z-score	Stunted	16/72	22.30 (12.85-42.27)	10/86	11.68 (6.64-22.60)	0.49 (0.21- 1.17)	0.89
	Not stunted	22/82	26.72 (16.75-45.36)	9/69	12.99 (5.37-39.95)	0.45 (0.19-1.06)	
Presence of a cough	No	28/139	20.16 (13.16-32.53)	17/143	11.92(7.09-21.68)	0.55 (0.29- 1.06)	0.2
	Yes	10/15	65.67 (37.38-126.21)	2/12	16.38 (4.10-65.50)	0.30 (0.06-1.68)	
Abnormal RR	No	10/79	12.66 (6.74-26.75)	11/95	11.55 (5.67-26.75)	0.81 (0.33-2.01)	0.19
	Yes	28/75	37.30 (24.59-59.40)	8/60	13.42 (6.50-32.48)	0.35 (0.15-0.82)	
History of TB treatment	No	27/119	22.74 (14.73-37.04)	9/101	8.94 (4.51-20.39)	0.42 (0.19-0.95)	0.71
	Yes	11/35	31.12 (16.92-63.72)	10/53	18.77 (8.88-46.93)	0.55 (0.20-1.44)	
Resistance to AZM at baseline	No	36/137	26.35 (18.25-39.49)	16/142	11.29 (6.56-21.17)	0.42 (0.22-0.81)	0.26
	Yes	2/17	11.47 (2.75-94.52)	3/13	22.82 (4.41-280.93)	1.38 (0.19-9.93)	
Season	Rainy	13/78	16.72 (9.68-31.56)	8/78	10.26 (5.33-22.48)	0.58 (0.23-1.45)	0.58
	Dry	25/76	32.75 (22.00-51.02)	11/77	14.30 (7.78-29.36)	0.42 (0.20-0.89)	
Calendar Period (years)	2016-2017	23/59	39.00 (26.66-59.29)	12/62	19.30 (10.03-42.25)	0.48 (0.22-1.05)	0.95
	2018-2019	15/95	15.78 (8.52-32.74)	7/95	7.40 (3.28-20.46)	0.46 (0.18-1.20)	

^1^events/ total person years

^2^per 100 person years

^3^adjusted for age (categorized as 6-10, 11-15 and 16+), sex, site, season, calendar time and HIV VL

^4^p-value from LRT.

## 4 Discussion

In this *post hoc* analysis of the BREATHE trial, we reported CD4 count <200 cells/mm^3^ and an abnormal respiratory rate at baseline were risk factors for AREs, with a two-fold higher rate of AREs among those with tachypnoea. Other studies have found tachypnoea to be strongly associated with HIV-associated CLD in children: a cross-sectional study from South Africa in 2005-2006 in children with HIV-associated CLD found that tachypnoea was associated with reduced FEV_1_, although their cohort was younger (mean age 5 years) and the predominant pathology was LIP, which is uncommon in the ART era [11]. An abnormal respiratory rate represents more severe CLD, which can in turn put patients at higher risk of respiratory exacerbations.

Advanced immunosuppression (i.e. aCD4 count <200 cells/mm^3^) was associated with a higher risk of AREs. This is a well-recognised factor for risk of infections including respiratory infections among individuals living with HIV [12]. We observed seasonal variation of respiratory illnesses, with ARIs being more common during the colder dry season in Zimbabwe [13]. This finding, also documented in other studies, is largely due to winter peaks of respiratory viruses including respiratory syncytial virus, which shows peak activity in June, July and August in Southern Africa and is one of the commonest causes of ARIs, both in SSA and globally [14]. Being recruited from Zimbabwe, compared to Malawi was also found to be a risk factor for AREs. There is no clear explanation for this. Possible reasons include differences in demographics or reporting. Poverty and the multiple associated risk factors have long been associated with lower respiratory tract infections [15]. According to the World Bank Poverty and Equity report, Zimbabwe has higher rates of national poverty at 70% compared to a national poverty rate of 51.5% in Malawi. The situation in Zimbabwe is also compounded by recent droughts and a deteriorating economic situation, which has made food security and health service delivery increasingly fragile [16], all of which may contribute to increased rates of AREs.

The BREATHE trial found that rates of total AREs were approximately 50% lower in the group who received weekly weight based dosing of azithromycin compared to the placebo group, and this association remained after adjusting for *a priori* determined factors [5]. In addition to trial arm, this *post hoc* analysis found that the crude rates of AREs differed by age, sex, site, FEV_1_ Z-score, CD4 count, HIV VL, being on second line ART, history of TB treatment, resistance to azithromycin at baseline, season and calendar year. Our findings show that azithromycin reduced AREs in participants with a baseline FEV_1_ Z-score ≥-2, (RR 0.25 95%CI 0.08-0.77). This result may also be related to previous AREs, and in turn possible baseline antibiotic resistance. A prospective cohort study from South Africa by Githinji *et al* found that amongst adolescents with HIV a lower FEV_1_ score was associated with previous lower respiratory tract infections [17]. These individuals may hence have had more frequent treatment with previous courses of antibiotics, possibly increasing the presence of baseline antibiotic resistance, making prophylactic azithromycin less effective in this subgroup. We also found azithromycin reduced AREs in participants with an abnormal respiratory rate, representing symptomatic CLD and a group who are more likely to experience recurrent AREs. These participants may hence benefit more from preventative weekly treatment with azithromycin. We also found variation in the effect of azithromycin with regards to resistance to azithromycin at baseline, but the test for interaction did not reach statistical significance (likely due to low statistical power); stratum specific rate ratios suggested increased rates of ARE among those resistant to azithromycin and reduced rates of ARE among those without azithromycin resistance at baseline (RR 1.38 95% CI 0.19-9.93 and RR 0.42 95% CI 0.22-0.81 respectively).

Azithromycin appeared to reduce AREs by 77% in males but only 23% in females (stratum specific RR in the azithromycin group was 0.23 and 0.77 respectively). One possible explanation for variation by sex, is engagement with care. In this cohort most participants were older adolescents and so would likely take responsibility themselves for accessing care. Being male and under the age of 30 is a risk factor for disengaging from HIV care [18] and HIV related mortality in SSA is higher in males for this reason [19]. In this cohort male adolescents may be less likely to seek acute care for AREs, or may only seek care with severe symptoms, resulting in an increased likelihood of experiencing AREs in the future. As a result preventative treatment may have a greater impact in this subgroup.

Notably, azithromycin is well tolerated, and has no significant interactions with commonly used ART [20]. The BREATHE trial reported that the number of adverse events was higher in the placebo arm than the azithromycin arm and only reported minor gastrointestinal side effects [5]. Prevention of AREs in this population is likely to have benefits that extend beyond reducing symptoms. AREs are linked to worsening FEV_1_ in both children and adults with cystic fibrosis [21], and in children with non-cystic fibrosis bronchiectasis pulmonary exacerbations are the main risk factor for a decline in FEV_1_ [22]. Prevention of AREs may hence protect against further decline in lung function. It is also important to consider the psychosocial impact of AREs. A cross sectional study in children with cystic fibrosis in the US found that children reporting an ARE in the previous 6 months had worse psychosocial scores on the child health questionnaire compared to those without a recent ARE, indicating that the impact of AREs goes beyond pure physical symptoms [23]. Frequent AREs also impact schooling due to missed school days. A study from Zimbabwe showed that children and adolescents with HIV miss more school days and are more likely to repeat a school year compared to their HIV negative peers [24]. This likely has a significant impact on educational attainment, hence any means of reducing this disparity should be a priority. However, when considering prophylactic use of azithromycin resistance is a key concern, and previous studies have shown emergence of resistance associated with use of azithromycin. A study investigating the effect of weekly azithromycin in indigenous children with non-cystic fibrosis bronchiectasis in Australia and New Zealand reported antibiotic resistance to be seven times higher in the treatment group compared to the placebo group [25]. A Cochrane systematic review on the use of macrolides in patients with cystic fibrosis found that 2 of the 10 included studies reported a significant increase in macrolide resistant strains of *Staphylococcus aureus* in patients receiving azithromycin [26]. This will not only remove the benefit of prophylactic azithromycin in this population, but is also an important public health concern.

To date no other studies have evaluated potential treatments for HIV associated CLD in children and adolescents, and none have looked at the incidence or risk factors for AREs in this population. Strengths of this study include its robust design: as a double blinded randomized placebo-controlled control trial it is best placed to evaluate the impact of azithromycin on ARE in this cohort, minimizing confounding and many potential sources of bias. However, this study also has important limitations. The definition of ARE was a clinical one and there may have been differences in ascertainment by trial site. However, a clinical definition was selected as a pragmatic approach as microbiological tests are often not available in these settings. The impact of azithromycin on AREs was a secondary outcome and the BREATHE trial was not powered for this outcome, as a result our findings provide a basis for further research rather than firm evidence. Additionally, residual confounding may still exist, for example this study did not examine smoking status, something which may be an important confounder, especially amongst older children. However, smoking was not considered by the trial group because of the negligible prevalence of smoking in this group reported in other studies [27,28]. This study also examined for a total of 14 risk factors and we acknowledge the limitations of multiple testing. [29]. Lastly this study focused on subgroup analyses and examined for effect modification using a statistical test for interaction. Tests for interaction are frequently underpowered and have known limitations [30]. As a result there is a risk we may have failed to detect effect modification where it does exist, and these results require cautious interpretation.

In summary**,** risk factors for AREs in this population included presence of an abnormal respiratory rate at baseline and having a baseline CD4 count <200/mm^3^. Azithromycin was effective at reducing AREs in male participants but there was no evidence among female participants. There was no statistical evidence for effect modification overall, however in stratified analyses there was evidence of azithromycin having a greater impact on reducing AREs in participants with chronic respiratory symptoms at baseline, those on 1^st^ line ART, those with a FEV_1_ score >-2 and participants without baseline resistance to azithromycin. This exploratory analysis has highlighted potential subgroups who may benefit the most from treatment with weekly azithromycin, although further evidence is required. The use of targeted treatment may reduce concerns regarding the emergence of AMR, and children and adolescents with HIV-associated CLD should be screened for these respiratory symptoms and clinical characteristics to help identify those most at risk of AREs and to guide management.

## Declaration of Competing Interest

Dr Ferrand is funded by the Wellcome Trust. Drs Rehman, and Simms are partly funded by grant MR/R010161/1 from the UK Medical Research Council (MRC) and the UK Department for International Development (DFID) under the MRC/DFID Concordat agreement. The remaining authors declare no conflict of interest.

## Appendix Supplementary materials

Image, application 1
