# Supplementary material for: Effect of azithromycin on incidence of acute respiratory exacerbations in children with HIV taking antiretroviral therapy and co-morbid chronic lung disease: a secondary analysis of the BREATHE trial
Source: eClinicalMedicine. 2021 Nov 13;42:101195. doi: 10.1016/j.eclinm.2021.101195 (PMC8599092; doi:10.1016/j.eclinm.2021.101195)
Supplement: Supplementary file 1 [file mmc1.docx]

**Supplementary Figures**

**Figure S1: Cumulative event curve by trial arm and sex**

**
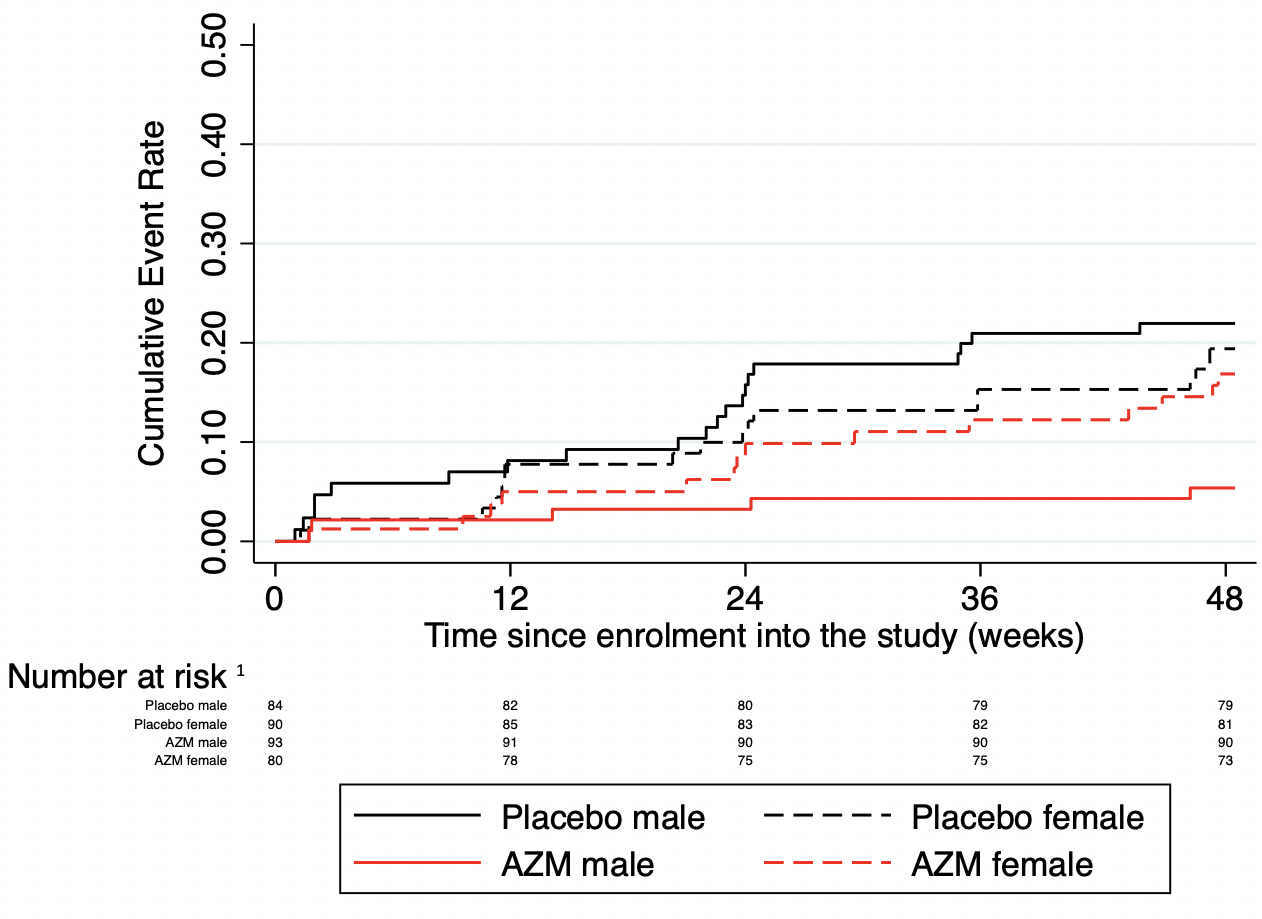
**

^1^Censored number are not recorded separately. Participants who experienced the event of interest (ARE) were retained in the at risk group (as participants can experience multiple AREs), so any decrease in the number of participants between time points is due to censoring only (from loss to follow up or death).

**Figure S2: Cumulative event curve by trial arm and FEV_1_ Z-score**

**Figure S3: Cumulative event curve by trial arm and ART line**

**Figure S4: Cumulative event curve by trial arm and presence of a cough at baseline**

**Figure S5: Cumulative event curve by trial arm and presence of an abnormal respiratory rate at baseline**

**Figure S6: Cumulative event curve by trial arm and resistance to AZM at baseline**
